# Association between inflammatory biomarkers and the cognitive response to a multidomain intervention: secondary longitudinal analyses from the MAPT study

**DOI:** 10.1007/s11357-024-01497-2

**Published:** 2025-01-17

**Authors:** Emmanuel Gonzalez-Bautista, Maria Soto, Gabor Abellan van Kan, Julien Delrieu

**Affiliations:** 1 Gerontopole, Clinical and Geroscience Research, Toulouse University Hospital, WHO Collaborating Center for Frailty, and Geriatric Training, Toulouse, France; 2 Maintain Aging Research Team, Centre d’Epidémiologie Et de Recherche en Santé Des POPulations, Université de Toulouse, Inserm, Université Paul Sabatier, Toulouse, France; 3 IHU HealthAge, Toulouse, France

**Keywords:** Multidomain intervention, Alzheimer’s disease, Inflammation, Cognitive composite score, GDF-15

## Abstract

The aim of this study is to evaluate the association of systemic inflammation measured by plasma biomarkers with the change in cognitive function among participants from the Multidomain Alzheimer Preventive Trial (MAPT) exposed to the multidomain intervention (MI). Secondary analysis of the MAPT longitudinal data. MAPT is a randomized, placebo-controlled trial with 3 interventional groups (omega-3 only, MI only, omega-3 plus MI) and a control group. We tested the association of the change in cognitive function with inflammatory biomarkers (tumoral necrosis factor receptor-1 (TNFR1), monocyte chemoattractant protein-1 (MCP1), Growth Differentiation Factor-15 (GDF15), Interleukin-6 (IL6) and C reactive protein (CRP)) using mixed-effects models. A subgroup analysis was performed in those exposed to the MI. The response to the MI was defined as the change in the composite cognitive score over the 2-year clinical follow-up period. by modeling the response to the intervention and identifying “good responders”, i.e., those in the 5th quintile of response at the end of the intervention period (2 years after the measurement of inflammatory markers). We included 1,527 participants (mean age 75.3, SD = 4.4; 64% female). Higher levels of GDF15 and TNFR1 were associated with a worse trajectory in the cognitive composite score in adjusted models. "Good responders" had an estimated mean change in the composite score of 0.051 (SD 0.062) over two years of intervention, compared to -0.136 (SD = 0.111) for the "not-good responders". Higher IL6 levels were associated with a decreased likelihood of being a "good responder" (OR = 0.22, *p* = 0.018, 95% CI 0.06; 0.78), with similar results for CRP (OR = 0.48, *p* = 0.009, 95% CI 0.28; 0.84). Higher inflammation was associated with a worse cognitive trajectory among nondemented participants and a lower likelihood of being classified as a "good responder" in those receiving a MI. Further confirmation of these findings could lead to the use of systemic inflammation as inclusion or stratification criteria in prevention trials.

**Supplementary Information:**

The online version contains supplementary material available at 10.1007/s11357-024-01497-2.

## Background

Multidomain interventions (MI) are strategies aimed at improving older people’s function or delaying its decline, and they involve at least two of the following: cognitive training, physical exercise, nutrition, or psychosocial components [1]. MI targeted to enhance cognitive capacity and slow cognitive decline has produced mixed results. A recent Cochrane review concluded that some features are repeatedly found in trials with positive results. (A) Endpoint measurement: there is a high certainty of evidence (GRADE) that MI provides a small but significative benefit against cognitive decline when measured with a neuropsychological test battery and compared to usual care/placebo (mean difference of a z-score = 0.03 SD higher in the MI group than in placebo or usual care) [2]. (B) Inclusion of cognitive training: it is associated with a positive effect, yet its impact on day-to-day functioning is arguable because participants might be just training on responding to the tests without any clinical relevance.

However, there is scarce information on how to target people with high chances of displaying a good response to MI preventing Alzheimer’s disease (AD) and cognitive decline trials. Older age, family history of AD, and mild cognitive impairment have been pointed out as features of participants at higher risk than the overall population, and thus, hypothetically having higher chances of being impacted by interventions [1, 3, 4]. Other studies have also included AD low-grade inflammation-related risk factors (e.g., cardiovascular risk factors like high blood pressure and HbA1c levels) among the inclusion criteria [1, 5]. Factors scores have been developed like the Cardiovascular Risk Factors, Aging and Dementia (CAIDE) [6] and Dementia Assessment Sheet for Community-based Integrated Care System 21-items (DASC-21), which include variables like age, level of education and hypertension, hypercholesterolemia, and obesity. MI studies have also found sub-group effects, for instance, in people with severe white matter hyperintensities [7]. Likewise, responses have been found among ApoE4 carriers [8].

Inflammatory cytokines are linked to cognition through a dual mechanism, wherein systemic cytokines signal the central nervous system (CNS) inflammation, and the systemic consequences of those cytokines ultimately affect cognition [9]. Cognitive manifestations of inflammatory damage appear when chronic excessive exposure to cytokines surpasses the functional cognitive reserve [9]. When activated, proinflammatory microglia can hinder the survival and growth of new cells and impact their integration into existing neural networks [10]. While neuroinflammation is strongly associated with cognitive impairments, there are still gaps in understanding the role of peripheral inflammation. However, peripheral cytokines can communicate with the central nervous system by stimulating vagal afferent nerves or brain vascular endothelial cells to produce proinflammatory cytokines, leading to symptoms such as impaired memory. In particular, systemic inflammation, vascular inflammation, and altered endothelial function may contribute to specific cognitive decline in individuals without dementia. Systemic inflammation, a risk factor for cardiovascular disease, can cross the blood–brain barrier and lead to responses causing neurodegeneration and cognitive impairment [11], for instance, IL-6, a marker of systemic inflammation, has been associated with cognitive decline and changes in brain structure[12].

A recent systematic review with metanalyses found that IL6 is among the most studied cytokines, with higher IL6 concentrations in AD patients compared to controls and in mild cognitive impairment (MCI) subjects compared to controls [9], and similar findings for TNFR-1 levels. Our group recently reported that the MI of Multidomain Alzheimer Preventive Trial (MAPT) positively improved cognition trajectories in participants with positive amyloid status defined by PET [10] or plasma Aβ 42/40 levels. [13] Further implication of inflammatory biomarkers has not been explored. The mechanisms by which multidomain interventions have improved cognitive decline are hypothesized to pass by stimulating cognition, nutrition, and locomotion and reducing risk factors; however, the molecular mechanisms are yet to be explored [1].

Characterizing inflammation levels in non-demented populations exposed to AD-prevention MI could help to select the best target population and increase the efficiency of the MI [14]. Thus, our objective was to evaluate the association of systemic inflammation measured by plasma biomarkers with the change in cognitive function among participants from the Multidomain Alzheimer Preventive Trial (MAPT) exposed to the multidomain intervention (MI).

## Methods

### Study

This is a secondary analysis of the MAPT study. The detailed methodology of MAPT has been described elsewhere [15, 16]. In summary, MAPT was a randomized controlled trial (RCT) testing the effect of a MI (nutritional counseling, physical exercise, and cognitive stimulation) with and without supplementation of omega-3 polyunsaturated fatty acids (omega-3) versus usual care on the prevention of cognitive decline among community-dwelling adults aged 70 years and older recruited in memory clinics in France. The Ethical Committee (CPP SOOM II) based in Toulouse approved the study (ClinicalTrials.gov identifier: NCT00672685). All participants signed a consent form before the study assessments. One blood sample of 15 ml (10 ml in an EDTA vacutainer and two 2.5 ml in PAXgene RNA tubes) was collected yearly for MAPT biobank. These samples were transferred directly at ambient temperature to the Cellular Biology and Cytology Laboratory at each site. The two PAXgene RNA tubes were frozen at − 20° directly. The EDTA tube was centrifuged then aliquoted; the serum and the pellet were collected in two 5-ml dry tubes, and then frozen at − 20°.

### Participants

MAPT inclusion criteria were meeting at least one of (a) spontaneous memory complaint expressed to their physician, (b) limitation in one instrumental activity of daily living (IADL), or (c) slow gait speed (≤ 0.8 m/s). Exclusion criteria comprised (a) participants with a Mini-Mental State Examination (MMSE) score < 24, (b) diagnosis of dementia, (c) limitation for one or more basic activities of daily living (ADLs), and (d) those taking omega-3 supplements at baseline. The selection of participants based on functional status was determined to target people with at least some degree of functional limitation (IADLs), hens with room to make improvements, but not yet touched in their ADLs, as this would be a proxy for care dependency [17].

The MI consisted of 2-h group sessions focusing on three domains (cognitive stimulation, physical activity, and nutrition) and a preventive consultation (at baseline, 12 months, and 24 months) with a physician to optimize management of cardiovascular risk factors and detect functional impairments [18]. Twelve small group sessions of the multidomain intervention were done in the first 2 months of the trial (two sessions per week in the first month, and one session per week in the second). Each session included 60 min of cognitive training (reasoning and memory training), 45 min of advice about and demonstrations of physical activity encouraging participants to increase their physical activity in their daily life to the equivalent of at least 30 min walking per day, 5 days a week, and was provided with a home-based program designed during individual interviews), and 15 min of nutritional advice based on French National guidelines. For the remainder of the 3-year study, participants in the multidomain intervention groups attended a 1-h session each month and two 2-h reinforcement sessions (12 and 24 months).

From the original 1679 participants enrolled in MAPT, we included participants with at least two available cognitive composite score measures and biomarker data (Supplementary Fig. 1). We defined the analytic start of our study by the time of the biomarker sampling (12 months after the MAPT study baseline) and worked with data for the rest of the intervention period. Sociodemographic and clinical data of interest for this study was collected at 12, 24, and 36 months after MAPT started.

### Composite cognitive score

The MAPT study used a cognitive composite score resulting from the z scores (using baseline means and standard deviations) of free and cued selective reminding test (FCSRT – free + total) for episodic memory [19], MMSE for orientation [20], Digit Symbol Substitution Subtest (DSST from Wechsler Adult Intelligence Scale-Revised) for attention and executive function [21], and Category Fluency Test for verbal fluency measured yearly within our study period. The MAPT composite score, similar to other indices of this kind, was derived from cohort studies in “normal controls” who progressed to mild cognitive impairment or Alzheimer dementia. Those studies determined that a composite measure sensitive to change in preclinical AD would likely require assessment of 3 key domains: episodic memory, executive function, and orientation [22]. The use of cognitive composite scores is supported by theoretical and empirical validations [23–25], and has been accepted by the FDA as a primary outcome in randomized clinical trials [26].

### Change in the cognitive composite score

First, using data from the overall MAPT population, we assessed the association of the change in the cognitive composite score and the plasma biomarkers adjusting for the MAPT allocation group (4 groups) in stepwise models.

### Response to the multidomain intervention

Secondly, we worked with the 531 participants randomized to the MI groups (with or without omega-3 supplementation).

We used two alternative definitions of the MI cognitive response: (i) those in the 5th quintile of change in the composite score in 2 years (20% best responders—this group can include people with a mild absolute negative change in the cognitive score) and (ii) participants with a change in cognitive composite score > 0 (only those with an improvement of the cognitive score over time).

### Inflammatory biomarkers

Plasma biomarkers selected as surrogates of peripheral inflammation were: IL6 [27, 28], TNFR1, MCP1 [29], GDF15, and CRP [30, 31]. Sample processing is described thoroughly in the supplementary material 1. All biomarker concentrations were measured in pg/mL except for CRP (mg/L) and were log transformed.

### Covariates

Models were adjusted for age, sex, education, apoE4 status (carrier versus non-carrier), clinical dementia rating (CDR) global score, MAPT allocation group (MI only or MI plus omega-3 supplementation) and intervention adherence (percentage of sessions attended) at baseline. Plasma Aβ 42/40 ratio was used as a covariate in the sensitivity analyses because it was only available on a subsample (*n* = 241).

### Statistical analyses

**Fig. 1:**
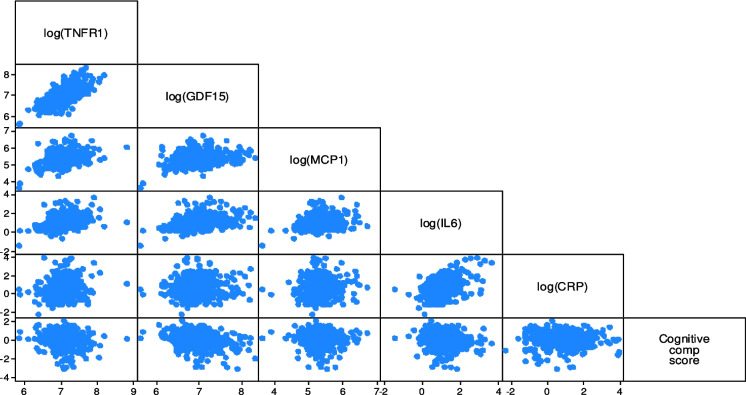
Graphical distribution of the baseline inflammation biomarkers and the cognitive composite score change during the study period. The scatterplots of the bottom line correspond to the unadjusted modeled associations in Table 2, which have the cognitive composite score in the y-axis and the biomarker log levels in the x-axis

We used means and standard deviations (SD) to describe continuous variables and frequencies and percentages for categorical/binary variables. We explored the distribution of the biomarkers and the cognitive response using scatterplots **(**Fig. 1).

We ran linear mixed models with a random intercept at the individual level to estimate the association of the biomarker levels with the change in the cognitive composite score over time: (A) adjusted for sex, age, and MAPT group (using the no-MI group as a reference), (B) adjusted for A + education, CDR score, ApoE4 status, and (C) B + plasma Aβ 42/40 ratio. These models take into account the individual-level variance, which adjusts for the starting cognitive score [32].

### Operational definitions of the MI cognitive response

(i) We predicted the individual trajectory of cognitive score over the study period (24 months) using mixed models for the time variable with random intercepts at the participant level and random slope for time (“empty model”). Then, we divided the slope of such trajectories into quantiles, the fifth quintile identifying the 20% best-off responders (steeper slope for composite cognitive score change over time). We coded a binary variable with “good responders (Q5)” = 1 and “other participants (Q1–Q4)” = 0. We ran logit models with this binary variable as the dependent and predictors as in models A, B, and C.

(ii) We used an alternative definition for “good responders”, including only the participants with a change in cognitive composite score > 0 (meaning their cognitive score improved over time) for our study period.

All analyses were evaluated at α = 0.05 and were performed using Stata 18. Stata Corp. 2023. College Station, TX: StataCorp LLC.

## Results

We included 1527 participants with at least two cognitive measures. Their mean age was 75.3 (standard deviation, SD = 4.3), and 64% were female. About 1 out of 4 participants (23.3%) were ApoE4 carriers (Table 1). Among those, 1089 had biomarker data available. The cognitive composite score showed a statistically significant monthly decrease of 0.0048 (95% CI − 0.0091; − 0.0005) per each log unit of TNFR1, and a statistically significant monthly decrease of 0.0049 (95% CI − 0.0085; − 0.0013) per each log unit of GDF-15, adjusting for age, sex, MAPT allocation group, education level, CDR score, and ApoE4 status. (**Model B **Table 2). The association of the cognitive composite score with the other biomarkers was not statistically significant. Adjusting for plasma Ab 42/40 ratio rendered the associations non-significant (*n* = 382).

**Table 1:** Description of the study population

	Not exposed to multidomain intervention expo	Exposed to multidomain intervention		Overall
		Participants with a cognitive change in Q1-Q4	Participants with a cognitive change in Q5 “good responders”	
*N*	842 (55.1%)	548 (35.9%)	137 (9.0%)	1527 (100.0%)
Age	75.4 (4.514)	75.5 (4.309)	73.9 (3.796)	75.3 (4.400)
Sex				
Female	548 (65.1%)	338 (61.7%)	92 (67.2%)	978 (64.0%)
Male	294 (34.9%)	210 (38.3%)	45 (32.8%)	549 (36.0%)
Cognitive composite score	0.137 (0.771)	0.103 (0.761)	0.314 (0.750)	0.141 (0.767)
CDR				
0	372 (53.4%)	255 (47.4%)	80 (58.8%)	707 (51.6%)
0.5	321 (46.1%)	282 (52.4%)	55 (40.4%)	658 (48.0%)
1	4 (0.6%)	1 (0.2%)	1 (0.7%)	6 (0.4%)
Education				
< 12 years	459 (56.4%)	314 (57.8%)	66 (48.5%)	839 (56.2%)
≥ 12 years	355 (43.6%)	229 (42.2%)	70 (51.5%)	654 (43.8%)
ApoE4 carrier				
No	506 (77.6%)	351 (75.6%)	92 (76.0%)	949 (76.7%)
Yes	146 (22.4%)	113 (24.4%)	29 (24.0%)	288 (23.3%)
BMI	26.3 (3.9)	26.4 (4.2)	25.7 (3.8)	26.3 (4.0)
MMSE (/40)	28.0 (1.9)	28.1 (1.8)	28.2 (1.7)	28.0 (1.9)
Frailty				
Robust	316 (51.0%)	256 (53.2%)	74 (62.2%)	646 (53.0%)
Pre_frail	281 (45.3%)	211 (43.9%)	44 (37.0%)	536 (43.9%)
Frail	23 (3.7%)	14 (2.9%)	1 (0.8%)	38 (3.1%)
Inflammatory biomarkers				
TNFR1*	1222.5 (415.6)	1253.5 (486.6)	1117.7(337.7)	1223.7 (439.1)
GDF15*	1120.7 (510.1)	1164.7 (532.7)	992.8 (384.0)	1124.5 (509.6)
MCP1*	221.6 (86.4)	223.1 (87.7)	220.1 (84.8)	222.1 (86.7)
IL6*	4.3 (17.0)	3.5 (3.2)	3.5 (4.5)	3.9 (12.3)
CRP [mg/L]	0.45 (1.7)	0.32 (1.4)	0.16 (0.8)	0.37 (1.5)

*BMI* body mass index, *MVPA* moderate-to-vigorous intensity physical activity, *MET* metabolic equivalent task, *ApoE4* Apolipoprotein E4, *IL6* Interleukin-6, *TNFR1* tumoral necrosis factor receptor-1, *MCP1* monocyte chemoattractant protein-1, *GDF15* Growth Differentiation Factor-15, *CRP C* reactive protein

^*^Unit [pg/mL]

**Table 2:** Mixed effect models for the change in cognitive composite score (continuous variable) in the overall MAPT population

	Model A				Model B				Model C			
	Age, sex and MAPT group				A + education, CDR, ApoE4				B + plasma Ab 42/40 ratio			
	Coeff	*p*	CI95%lb	CI95%ub	Coeff	*p*	CI95%lb	CI95%ub	Coeff	*p*	CI95%lb	CI95%ub
*n*	1089				968				382			
TNFR1	** − 0.0045**	**0.033**	** − 0.0086**	** − 0.0004**	** − 0.0048**	**0.0280**	** − 0.0091**	** − 0.0005**	** − **0.0067	0.0930	** − **0.0145	0.0011
GDF15	** − 0.0049**	**0.005**	** − 0.0083**	** − 0.0015**	** − 0.0049**	**0.0070**	** − 0.0085**	** − 0.0013**	** − **0.0056	0.1070	** − **0.0124	0.0012
MCP1	** − **0.0005	0.789	** − **0.0043	0.0033	** − **0.0007	0.7160	** − **0.0048	0.0033	** − **0.0010	0.8070	** − **0.0089	0.0069
IL6	0.0005	0.657	** − **0.0016	0.0025	0.0003	0.7650	** − **0.0018	0.0025	** − **0.0026	0.2140	** − **0.0067	0.0015
CRP	0.0002	0.927	** − **0.0048	0.0053	0.0019	0.5290	** − **0.0039	0.0077	0.0031	0.5100	** − **0.0062	0.0124

Coeff. = the estimated change in the cognitive composite score by per month, by each increase in one unit of the log transformed biomarker

*ApoE4* Apolipoprotein E4, *IL6* Interleukin-6, *TNFR1* tumoral necrosis factor receptor-1, *MCP1* monocyte chemoattractant protein-1, *GDF15* Growth Differentiation Factor-15, *CRP* C reactive protein

Statistically significant results are marked in bold

### MAPT participants exposed to the MI

Among MAPT participants exposed to the MI (with or without omega-3 supplementation), “good responders” were younger and had better CDR scores than their counterparts at baseline **(**Table 1**)**. They had an estimated mean change in the composite score of 0.051 (SD 0.062) over 2 years of intervention, compared to − 0.136 (SD = 0.111) for the not-good responders.

For each log unit increase in GDF-15, the cognitive composite score decreased significantly 0.0053 (95% CI − 0.0105; − 0.0002) points monthly, adjusting for age, sex, education, CDR score, ApoE status, MAPT allocation group, and adherence to the MI **(**Table 3** Model B)**. No other significant associations were found for models A and B. Among participants with available data for plasma Ab 42/40 ratio (*n* = 187), a significant decrease in the cognitive composite score was found for each log unit increase in TNFR1 (− 0.0157, 95% CI − 0.0279; − 0.0034) and also for GDF-15 (− 0.0116, 95% CI − 0.0215; − 0.0016) **(**Table 3** Model C).**

**Table 3:** Mixed effect models for the change in cognitive composite score (continuous variable) in participants exposed to the MI

	Model A				Model B				Model C			
	Age and sex				A + education, CDR, ApoE4, MAPT group and adherence				B + plasma Aβ 42/40 ratio			
	Coeff	*p*	CI95%lb	CI95%ub	Coeff	*p*	CI95%lb	CI95%ub	Coeff	*p*	CI95%lb	CI95%ub
*n*	531				478				187			
TNFR1	− 0.0043	0.180	− 0.0105	0.0020	− 0.0060	0.068	− 0.0124	0.0004	** − 0.0157**	**0.012**	** − 0.0279**	** − 0.0034**
GDF15	− 0.0039	0.121	− 0.0089	0.0010	** − 0.0053**	**0.042**	** − 0.0105**	** − 0.0002**	** − 0.0116**	**0.022**	** − 0.0215**	** − 0.0016**
MCP1	0.0008	0.772	− 0.0046	0.0063	0.0004	0.883	− 0.0053	0.0062	− 0.0050	0.417	− 0.0172	0.0071
IL6	0.0018	0.249	− 0.0013	0.0048	0.0015	0.362	− 0.0017	0.0047	− 0.0048	0.174	− 0.0117	0.0021
CRP	0.0009	0.359	− 0.0010	0.0028	0.0008	0.430	− 0.0012	0.0028	− 0.0007	0.679	− 0.0042	0.0027

Coeff. = the estimated change in the cognitive composite score by per month, by each increase in one unit of the log transformed biomarker

*ApoE4* Apolipoprotein E4, *IL6* Interleukin-6, *TNFR1* tumoral necrosis factor receptor-1, *MCP1* monocyte chemoattractant protein-1, *GDF15* Growth Differentiation Factor-15, *CRP* C reactive protein

Statistically significant results are marked in bold

### Odds of being in the good responder group (logit models)

Higher plasma levels of GDF15 and TNFR1 were significantly associated with lower chances of being in the cognitive “good responder” group when adjusting for age and sex. However, this association remained significant only for TNFR1 after adjusting for education, CDR baseline status, ApoE4, MAPT group, and adherence. For each log increase in TNFR1, the odds of being in the “good responder” group lowered by 59% (OR = 0.41, 95% CI 0.18; 0.94) **(**Table 4**).** In other words, for each increase in one log unit of the TNFR1, the odds of being in the “good responder” group decreased by 34% compared to those in the non-good responders’ group.

**Table 4:** Logit models for the odds of being in the “good responders” group compared to being in the “other participants”

	Model A				Model B				Model C			
	Age and sex				A + education, CDR, ApoE4, MAPT group and adherence				B + plasma Aβ 42/40 ratio			
	OR	*p*	CI95%lb	CI95%ub	OR	*p*	CI95%lb	CI95%ub	OR	*p*	CI95%lb	CI95%ub
*n*	531				478				187			
TNFR1	0.44	0.031	0.20	0.93	0.41	0.036	0.18	0.94	0.36	0.312	0.05	2.61
GDF15	0.49	0.033	0.26	0.94	0.55	0.110	0.27	1.14	0.73	0.734	0.11	4.62
MCP1	1.10	0.767	0.59	2.03	1.12	0.749	0.57	2.21	1.05	0.948	0.24	4.55
IL6	0.95	0.772	0.67	1.35	1.01	0.978	0.68	1.49	0.22	0.018	0.06	0.78
CRP	0.97	0.798	0.79	1.20	0.98	0.861	0.77	1.24	0.48	0.009	0.28	0.84

OR = the odds ratio of being in the “good responders” group compared to being among the “no good responders” per each increase in one unit of the log transformed biomarker

*ApoE* Apolipoprotein E, *IL6* Interleukin-6, *TNFR1* tumoral necrosis factor receptor-1, *MCP1* monocyte chemoattractant protein-1, *GDF15* Growth Differentiation Factor-15, *CRP* C reactive protein

Similar results were found with the second definition of good responders (absolute positive change in the composite score over time). In the model adjusted for age, sex, education, CDR, ApoE4, MAPT group, and adherence (*n* = 478), the possibility of being a good responder was OR = 0.41, 95% CI 0.18 for each unit increase in the log transformed TNFR1 (Supplementary Table S1).

Participants with higher IL6 or CRP levels showed significantly lower odds of being in the “good responder” group **(Model C **Table 4**).** For each log increase in IL6 levels, the odds of being in the “good responder” group decreased by 78% (OR = 0.22, *p* = 0.018, 95% CI 0.06; 0.78), likewise, those odds decreased by 52% for each log increase in CRP (OR = 0.48, *p* = 0.009, 95% CI 0.28; 0.84). No association was found for MCP1.

## Discussion

The levels of inflammatory plasma biomarkers (TNFR1 and GDF15) were inversely and significantly associated with the change in cognitive function within 2 years of the second cognitive measure in MAPT participants. Among those exposed to the MI, this association was independent from the participants’ level of plasma Aβ 42/40 ratio and ApoE4 status. Moreover, participants who showed a good response to the MI (i.e., the 20% best-off of change in cognitive function), registered low peripheral inflammation biomarkers (TNFR1, GDF15, IL6, and CRP) within 2 years of the second cognitive measure, independently from their Aβ 42/40 ratio levels and ApoE4 status. When adjusting for plasma amyloid levels, higher IL6 and CRP plasma levels were significantly associated with lower odds of being in the “good responder” group.

The impact of MI on global cognition has been inconclusive so far [33]. Meta-analyses indicate that multidomain interventions have small but significant effects on reducing dementia risk and improving cognitive composite scores, although effects on global cognition remain inconclusive [34]. Large RCTs have found negative primary outcome results, but some significant findings when looking at participants with higher dementia risk [7, 18].

To our knowledge, this is the first study to identify peripheral inflammation associated with a good MI response in nondemented older adults with memory complaints.

Previous works have identified a positive response to MI among ApoE4 carriers and positive amyloid participants [13, 35]. These two biomarkers are related to the AD pathophysiology. They thus might indicate the impact of MI as secondary prevention in people who are at risk for developing AD or at an early symptomatic stage of the disease. However, according to our results, we think that MI might also exert an effect on cognitive function in non-AD patients with memory complaint afflicted by other cognitive deteriorations related to aging and inflammation. In other words, by selecting participants with low inflammatory markers, clinical trials, and population-based interventions could open their scope to the primary prevention of cognitive decline for both AD and non-AD patients without systemic inflammation. As can be seen in Supplementary Figure S2, plasma levels of GDF15, IL6, and CRP are consistently low in “good responders”, regardless of their amyloid status.

The association between systemic inflammation and cognitive decline has been studied during the last two decades. This link has been found in middle-aged [36] and older subjects [37]. In AD pathogenesis, inflammation has been suggested to play a role in amyloid brain deposition [38]. Put another way, finding low inflammatory levels in people with memory complaints might be a marker of good cognitive reserve [9] or a “fertile field” to receive an MI. A complex cross-talk between the immune and neurological systems involves cytokines as modulators of the neuronal and glial cell function in neuronal regeneration or neurodegeneration [39].

Our study has strengths. We have used data from a clinical trial, which allows for a thorough measurement of the clinical outcome after a standardized exposure and adjusting by adherence to the intervention. Additionally, we were able to control for the effect of previously explored variables related to the outcome, like ApoE4 and amyloid status. This allowed us to bridge further the knowledge gap on factors associated with a MI. Our study had limitations; for instance, we could not describe the trajectory of the inflammatory biomarkers along the intervention because they were measured only once. The biomarker sampling did not coincide with the beginning of the MAPT study but 12 months after, which impeded us from including the complete intervention period (initially 3 years). However, had the biomarker level lowered due to the intervention during the first 12 months, this effect would have reinforced the association we found (lower biomarker levels linked to higher cognitive response). Also, our sample size was affected, given that plasma Aβ 42/40 ratio was only measured in a small subsample of participants.

This study does not allow for detailed evaluation of each intervention individually; particularly, given that the MAPT study was designed as a 1:1:1:1 randomized controlled trial. Furthermore, given that the omega-3 supplementation without MI has not shown a positive effect in older adults’ cognition trajectories, we focused our research question on the response to the MI.

### Perspectives

In future studies, we foresee the instrumentation of a biochemical profiling of patients who are candidates for a MI. Using a panel of plasma biomarkers can be an innovative step forward to personalized prevention of cognitive decline. Additionally, AD and neurodegeneration blood biomarkers [40], including inflammation, could be helpful to reach a more efficient population in cognitive decline prevention trials.

## Conclusion

“Good responders” to the MAPT MI showed lower GDF15 and TNFR1 baseline blood levels than their counterparts. MAPT participants who increased their cognitive score after 2 years of intervention had lower IL6 and CRP baseline levels, regardless of their Aβ 42/40 and ApoE4 status. More research is needed to describe the trajectories of inflammation along the MI. Also, further trials should explore if inflammation-lowering interventions like physical activity, diet, or anti-inflammatory drugs should be implemented before or concomitantly with cognitive stimulation in MI. Using inflammatory biomarkers as a selection criterion for AD primary preventive trials might lead to a more efficient enrolling process for the research and real-world clinical contexts.

## Supplementary Information

Below is the link to the electronic supplementary material.

ESM 1 (DOCX 17.1 KB)

## Data Availability

MAPT data is available upon request and validation from the MAPT DSA team. Info.u1027-dsa@inserm.fr.
