# Supplementary material for: Association between inflammatory biomarkers and the cognitive response to a multidomain intervention: secondary longitudinal analyses from the MAPT study
Source: GeroScience. 2025 Jan 17;47(3):5365–76. doi: 10.1007/s11357-024-01497-2 (PMC12181457; doi:10.1007/s11357-024-01497-2)
Supplement: Supplementary file 1 — (DOCX 17.1 KB) [file 11357_2024_1497_MOESM1_ESM.docx]

**Supplementary material**

**Supplementary Figure 1. Flow of participant sample size**

**Table S1. Logit models for the odds of being in the "good responders" group compared to being in the "other participants" according to the alternative definition for "good responders" based on having a positive change for the cognitive composite score**

|  | A) Age and sex | | | |  | Model B  A + education, CDR, ApoE4, MAPT group and adherence | | | |  | Model C  B + plasma Aβ 42/40 ratio | | | |
| --- | --- | --- | --- | --- | --- | --- | --- | --- | --- | --- | --- | --- | --- | --- |
|  | OR | p | CI95%lb | CI95%ub |  | OR | p | CI95%lb | CI95%ub |  | OR | p | CI95%lb | CI95%ub |
| n | 531 |  |  |  |  | 478 |  |  |  |  | 187 |  |  |  |
| TNFR1 | **0.44** | **0.033** | **0.21** | **0.94** |  | **0.41** | **0.038** | **0.18** | **0.95** |  | 0.36 | 0.312 | 0.05 | 2.61 |
| GDF15 | **0.52** | **0.047** | **0.27** | **0.99** |  | 0.59 | 0.153 | 0.28 | 1.22 |  | 0.73 | 0.734 | 0.11 | 4.62 |
| MCP1 | 1.11 | 0.737 | 0.60 | 2.06 |  | 1.14 | 0.702 | 0.58 | 2.27 |  | 1.05 | 0.948 | 0.24 | 4.55 |
| IL6 | 0.97 | 0.882 | 0.69 | 1.38 |  | 1.04 | 0.858 | 0.70 | 1.53 |  | **0.22** | **0.018** | **0.06** | **0.78** |
| CRP | 0.97 | 0.808 | 0.79 | 1.21 |  | 0.98 | 0.850 | 0.77 | 1.24 |  | **0.48** | **0.009** | **0.28** | **0.84** |

OR = the odds ratio of being in the "good responders" group compared to being among the "no good responders" per each increase in one unit of the log transformed biomarker.

Apolipoprotein E4 (ApoE4), Interleukin-6 (IL6), Tumoral necrosis factor receptor-1 (TNFR1), Monocyte chemoattractant protein-1 (MCP1), Growth Differentiation Factor-15 (GDF15), C reactive protein (CRP).

**Figure S2. Distribution of inflammatory biomarkers among good responders and other participants to the MAPT multidomain intervention by amyloid status**

**Amyloid positive status was defined based on plasma Aβ 42/40 ratio > 0.107 pg/mL**

**Supplementary material S3**

Detailed biomarker analyses

Interleukin-6 (IL-6), tumor necrosis factor receptor-1 (TNFR-1), monocyte chemoattractant protein-1 (MCP-1), and growth differentiation factor-15 (GDF-15) were assessed using the fully automated immunoassay platform, Ella (ProteinSimple/Bio-techne, San Jose, CA, USA). Proteins were quantified using a single disposable microfluidic SimplePlexTM cartridge. The plasma samples were thawed on ice, diluted 1:4 in sample diluent (SD 13), and loaded into cartridges with relevant high and low control concentrates. Each plasma sample was divided into four unique microfluidic parallel channels within the cartridge, specific for each of the four proteins being analyzed. Each protein channel contains three analyte-specific glass nanoreactors (GNRs), allowing each plasma sample to be run in triplicates for each of the four protein samples. Cartridges included a built-in lot-specific standard curve for each defined protein. The instrument automatically conducted all the procedure steps without user activity. The obtained protein concentrations were calculated by the internal instrument software (unit: pg/mL). CRP was measured (mg/L) by immunoturbidity according to standard protocols.

AD biomarkers were assessed at the 12-month visit. Plasma samples were spiked with a known quantity of 15N-Aβ42 and 15N-Aβ40 for use as analytical internal standards. Aβ42 and Aβ40 isoforms were simultaneously immunoprecipitated from 0.45 mL of plasma via a monoclonal anti-Aβ mid-domain antibody (HJ5.1, anti-Aβ13-28) conjugated to M-270 Epoxy Dynabeads (Invitrogen). LysN endoprotease (Pierce) was used for protein digestion into peptides. Liquid chromatography-mass spectrometry was performed as detailed by Schindler et al. 2019. Plasma analyses were performed as targeted parallel reaction monitoring on an Orbitrap Fusion Lumos Tribrid mass spectrometer (Thermo Fisher) interfaced with an M-class nanoAcquity chromatography system (Waters). Derived integrated peak areas were analyzed using the Skyline software package. Aβ42 and Aβ40 were quantified by integrated peak area ratios to known concentrations of the internal standards. Plasma Aβ42/40 ratio was then determined by dividing Aβ42 by Aβ40, and its normalized values were used.

Reference

Schindler SE, Bollinger JG, Ovod V, Mawuenyega KG, Li Y, Gordon BA, Holtzman DM, Morris JC, Benzinger TLS, Xiong C, Fagan AM, Bateman RJ. High-precision plasma β-amyloid 42/40 predicts current and future brain amyloidosis. Neurology. 2019;93:e1647–59. https://doi.org/10.1212/WNL.0000000000008081 .
